# The Pipeline of Therapeutics Testing During the Emergency Phase of the COVID-19 Outbreak

**DOI:** 10.3389/fmed.2020.552991

**Published:** 2020-09-24

**Authors:** Marco Canevelli, Giulia Remoli, Federica Trentin, Gabriele Riccardi, Leonardo Tariciotti, Giovanni Risoleo, Antonio Ancidoni, Giuseppe Bruno, Matteo Cesari, Nicola Vanacore, Valeria Raparelli

**Affiliations:** ^1^National Center for Disease Prevention and Health Promotion, Italian National Institute of Health, Rome, Italy; ^2^Department of Human Neuroscience, Sapienza University of Rome, Rome, Italy; ^3^Department of Sense Organ, Sapienza University of Rome, Rome, Italy; ^4^Unit of Neurosurgery, IRCSS Fondazione Cà Granda Ospedale Maggiore Policlinico, University of Milan, Milan, Italy; ^5^Department of Radiology, ASTT Grande Ospedale Metropolitano Niguarda, University of Milan, Milan, Italy; ^6^Department of Clinical Sciences and Community Health, University of Milan, Milan, Italy; ^7^Geriatric Unit, IRCCS Istituti Clinici Scientifici Maugeri, Milan, Italy; ^8^Department of Experimental Medicine, Sapienza University of Rome, Rome, Italy

**Keywords:** COVID-19, SARS-CoV-2, clinical trials, antivirals, immunomodulators, research protocols, drug development

## Abstract

The coronavirus disease 19 (COVID-19) pandemic poses a serious threat to the sustainability of healthcare systems and is currently having a significant effect on living conditions worldwide. No therapeutic agent has yet proven to be effective for the treatment of COVID-19. The management of this disease currently relies on supportive care and the off-label and compassionate use of antivirals and immunomodulators. Nevertheless, there has been a great worldwide effort to progress research and test the efficacy and safety/tolerability profiles of numerous candidate agents that may positively affect the various clinical syndromes associated with COVID-19. In parallel, vaccination and chemoprophylaxis strategies are being investigated. This article provides a summary of interventional studies targeting COVID-19 during the emergency phase of the outbreak to broadly inform clinicians and researchers on what happened and what they can expect in upcoming months. The clinicaltrials.gov database and the European Union (EU) Clinical Trials Register were investigated on March 31, 2020, to identify all ongoing phase 1–4 research protocols testing pharmacological interventions targeting SARS-CoV-2 infection and/or clinical syndromes associated with COVID-19. Overall, six phase 1, four phase 1-2, 14 phase 2, ten phase 2-3, 19 phase 3, and nine phase 4 studies were identified, and the features of these studies are described in the present review. We also provide an updated overview of the change overtime in the pipeline following this emergency phase and based on the current epidemiology of the COVID-19 pandemic.

## Introduction

The coronavirus disease 19 (COVID-19) pandemic has been caused by the severe acute respiratory syndrome coronavirus 2 (SARS-CoV-2). It poses a serious threat to the sustainability of healthcare systems, with substantial effects on living conditions worldwide. As of April 3, 2020, more than one million COVID-19 cases and around 53,000 deaths have been calculated in 181 countries worldwide (1). In parallel, nearly half of the global population is currently in lockdown.

To date, no therapeutic compound has been proven to be effective for the treatment of COVID-19. In the initial emergency phase of the outbreak, therapeutic management of affected individuals relied on supportive care (2, 3) and on the off-label and compassionate use of a variety of antiviral (e.g., lopinavir/ritonavir, remdesivir, favipiravir) and/or immunomodulator (e.g., chloroquine, hydroxychloroquine, anti-IL-6 inhibitors, steroids) drugs, the efficacy of which had not then been demonstrated (4, 5). Moreover, their safety and tolerability profiles in patients with COVID-19 remains to be clarified (4, 6).

In this pandemic scenario, a great deal of effort is currently being devoted to the identification of novel therapies and prophylactic strategies, with new research protocols registered internationally every week (if not daily) (7). Moreover, the urgent need to move this field forward in response to this ongoing outbreak needs to be counterbalanced by ensuring that the products under investigation are evaluated through scientifically and ethically appropriate studies (8). There are challenging time-frames connected to the process of developing new therapeutic strategies against COVID-19 or repositioning existing compounds with plausible modifying effects on the disease. The clinical course of patients is not yet fully elucidated (9), and there is incomplete data on the underlying pathophysiological mechanisms (10) and potential therapeutic targets.

In this article, we provide a summary of the interventional studies that have been conducted worldwide to test the efficacy and/or safety/tolerability of pharmacological compounds against COVID-19 in the emergency phase of the pandemic.

## Methods

### Data Source

Two databases, the clinicaltrials.gov database and the European Union (EU) Clinical Trials Register, constituted the reference sources for the present study. Clinicaltrials.gov is a web-based resource maintained by the US National Library of Medicine and the National Institute of Health that provides information on publicly and privately supported clinical studies. Registration on this database is mandatory for all clinical investigations of any US Food and Drug Administration (FDA)-regulated drug or medical device. However, it also represents a repository for the vast majority of clinical trial protocols conducted worldwide. EU Clinical Trial Register gathers information on ongoing authorized interventional studies in the EU and the European Economic Area (EEA) that are registered in the EU Drug Regulation Authorities Clinical Trials Database (EudraCT).

### Search Strategy

The databases were investigated on March 31, 2020, using the following search terms: “COVID-19” OR “SARS-CoV-2” OR “2019 novel coronavirus” OR “2019-nCoV” OR “severe acute respiratory syndrome coronavirus 2” OR “coronavirus.” In clinicaltrials.gov, the advanced search function was used to restrict the search to: (i) interventional studies (STUDY TYPE); (ii) “recruiting,” “enrolling by invitation,” and “active not recruiting” protocols (STATUS: RECRUITMENT); and (iii) phase 1, phase 2, phase 3, phase 4 studies (PHASE).

**Figure 1:**
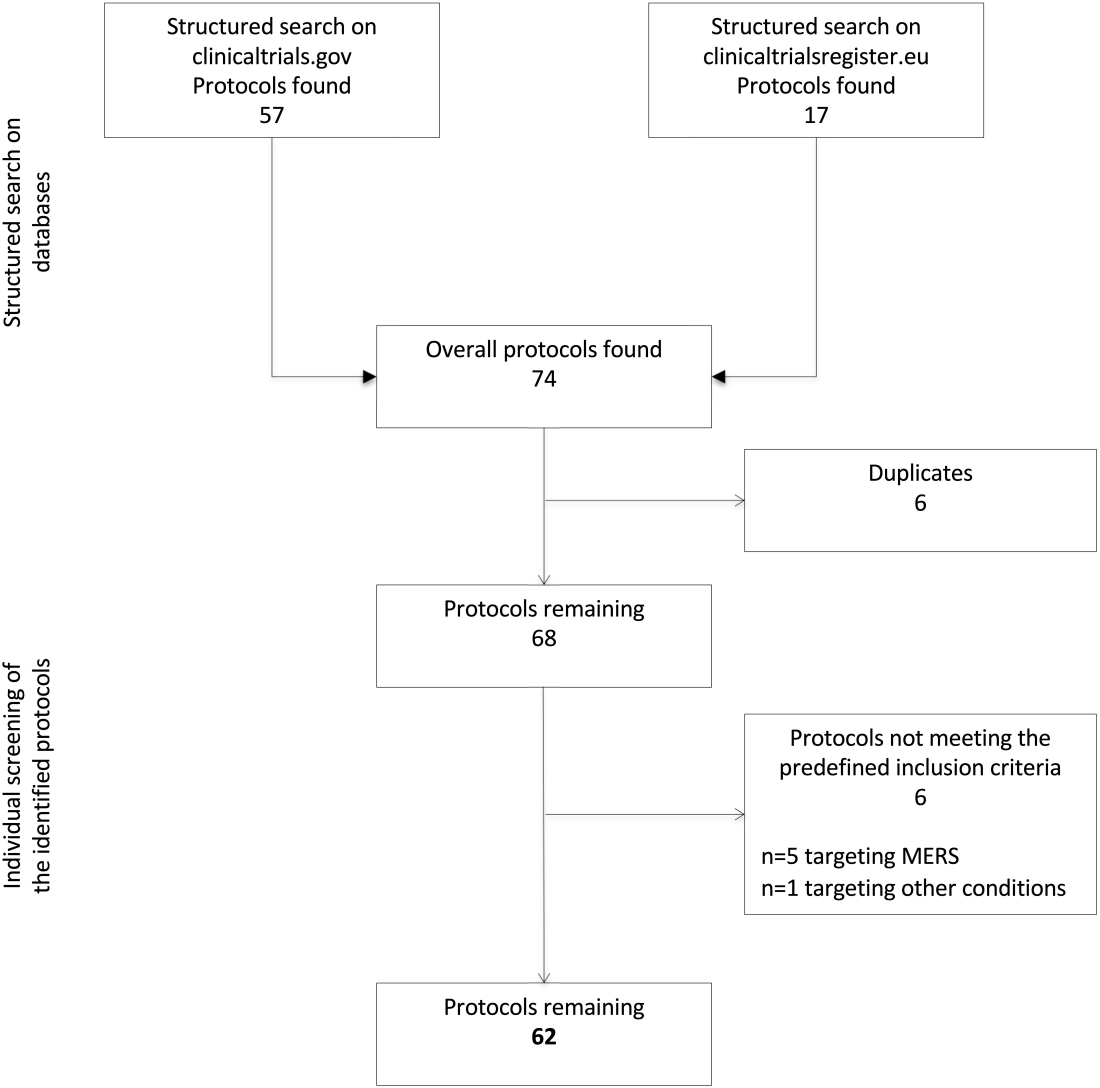
Flow-chart describing the selection of protocols. MERS, Middle East Respiratory Syndrome.

Two reviewers (L.T. and G.R.) screened the identified protocols to remove duplicates and verify the fulfillment of the following predefined inclusion criteria: (1) targeting SARS-CoV-2 infection and/or clinical syndromes associated with COVID-19; and (2) testing the efficacy and/or safety/tolerability of pharmacological interventions. Studies investigating novel medical devices or diagnostic tools were not considered in the present analysis. Disagreements in the selection were solved by consensus, involving two additional reviewers (M.C. and V.R.). The flow chart in Figure 1 illustrates the process of protocols' selection.

### Data Extraction

The following data were abstracted by three authors (F.T., Ga.R., and Gi.R.) from the selected protocols: NCT (the unique identification code assigned by clinicaltrials.gov) and/or EudraCT codes; study phase; allocation and masking procedures; tested compound(s); way of administration; mechanism of action; primary outcome measure(s); expected primary completion date; expected number of participants; age range of participants; targeted COVID-19 related condition; sponsor; and location.

Clinical syndromes associated with COVID-19 were coded according to the classifications provided by the World Health Organization (WHO) (i.e., mild illness, pneumonia, severe pneumonia, acute respiratory distress syndrome [ARDS], sepsis, and septic shock) (2). When the WHO classification could not be applied or was not specified, the targeted conditions were classified according to the definitions provided in the protocol.

## Results

### Search Results

A total of 74 protocols were identified through a structured search of the two adopted databases (*n* = 57 on clinicaltrials.gov and *n* = 17 on EU Clinical Trials Register). After the removal of six duplicates, six additional protocols were excluded because not targeting COVID-19. Specifically, five of them were focused on the Middle East Respiratory Syndrome and one on other bacterial or viral infections. Thus, 62 protocols were ultimately retained. The reviewers reported a >90% agreement in the selection process.

### Characteristics of the Selected Protocols

Clinical trials involving new drugs are commonly classified into four phases, with some individual trials encompassing more than one phase (e.g., combined phase 1–2). Overall, six phase 1, four phase 1–2, 14 phase 2, ten phase 2–3, 19 phase 3, and nine phase 4 studies were identified. Their detailed characteristics are presented in Tables 1–**3**.

**Table 1:** Characteristics of the selected phase 1, phase 1-2, phase 2, and phase 2-3 protocols targeting COVID-19-related conditions.

**Identification Trial Number**	**Treatment(s) Comparator(s)(if any)**	**Primary completion**	**Allocation Assignment**	**Masking**	**Primary outcome(s)**	**Subjects**	**Age**	**Condition**
Phase 1								
NCT04252118	1. Mesenchymal stem cells IV 2. Conventional treatment	December 20	Non-randomized Parallel	None	- Size of lesion area by chest radiograph or CT (day 0,3,6,10,14,21,28) - Side effects (day 0,3,6,10,14,21,28,90,180)	20	18-70y	Pneumonia^*^
NCT04313322	1. Wharton's Jelly mesenchymal stem cells IV	June 20	Single group	None	- Improvement of clinical symptoms (week 3) - Side effects measured by chest radiograph (week 3) - RT-PCR results (week 3)	5	≥18y	Infection^*^
NCT04299724	1. Artificial antigen presenting cells (aAPC) vaccine SC	July 23	Single group	None	- Vaccine events and severe events (days 0-28) - Proportion of subjects with positive T cell response (days 0-28)	100	6m-80y	Healthy subjects Infection^*^
NCT04313127	1. Ad5-nCoV vaccine (low) IM 2. Ad5-nCoV vaccine (middle) IM 3. Ad5-nCoV vaccine (high) IM	December 20	Non-randomized Sequential	None	- Safety indexes of adverse reactions (days 0-7)	108	18-60y	Healthy subjects
NCT04283461	1. mRNA-1273 vaccine (low) IM 2. mRNA-1273 vaccine (middle) IM 3. mRNA-1273 vaccine (high) IM	June 21	Non-randomized Sequential	None	- Frequency of adverse events and new-onset chronic medical conditions (days 0-394)	45	18-55y	Healthy subjects
NCT04280224	1. Natural killer cells 2. Conventional treatment	September 20	Randomized Parallel	None	- Improvement of clinical symptoms (days 0-28) - Adverse events (days 0-28)	30	18-65y	Pneumonia^*^
Phase 1-2								
NCT04288102	1. Mesenchymal stem cells IV 2. Placebo	December 20	Randomized Parallel	Yes (PCIOa)	-Size of lesion area and severity of pulmonary fibrosis by chest CT (day 0,6,10,14,28,90)	90	18-75y	Severe pneumonia ARDS
NCT04324996	1. Natural killer (NK) cells IV 2. IL15-NK cells IV 3. NKG2D CAR-NK cells IV4. ACE2 CAR-NK cells IV5. NKG2D-ACE2 CAR-NK cells IV	May 20	Randomized Parallel	Yes (PCIOa)	- Clinical response (day 28) - Side effects (day 28)	90	≥18y	Severe pneumonia ARDS Sepsis/septic shock
NCT04276896	1. Synthetic minigene vaccine (LV-SMENP-DC) IV SC	July 23	Single group	None	- Clinical improvement (day 28) - Lower Murray lung injury score (day 7)	100	6m-80y	Healthy subjects Infection^*^
NCT04275245	1. Meplazumab IV	December 20	Single group	None	- Virological clearance rate using RT-PCR (day 3,7,14)	20	18-75y	Pneumonia^*^
Phase 2								
NCT04307693	1. Lopinavir/Ritonavir O 2. Hydroxychloroquine O 3. No intervention	May 20	Randomized Parallel	None	- Viral load (day 3,5,7,10,14,18)	150	16-99y	Pneumonia
NCT04280588	1. Fingolimod O 2. No intervention	July 20	Non-randomized Parallel	None	- Change of pneumonia severity on X-ray images (day 5)	30	18-85y	Pneumonia Severe pneumonia
NCT04317092EudraCT-2020-001110-38	1. Tocilizumab IV	December 20	Single group	None	- Mortality rate (month 1)	330	All	Severe pneumonia ARDS
NCT04279197	1. Fuzheng Huayu O 2. Placebo	December 22	Randomized Parallel	Yes (PI)	- Evaluation of pulmonary fibrosis (CT)(week 24) - Evaluation of lung function improvement (week 24)	136	18-65y	Pulmonary fibrosis^*^
NCT04305457	1. Nitric oxide IN 2. No intervention	April 21	Randomized Parallel	None	- Reduction in the incidence of patients requiring intubation and mechanical ventilation (day 28)	240	≥18y	Pneumonia Severe pneumonia
NCT04306393	1. Nitric oxide IN 2. No intervention	March 21	Randomized Parallel	Yes (P)	- Change of arterial oxygenation (48 hours)	200	18-99y	ARDS
NCT04269525	1. Umbilical cord derived mesenchymal stem cells IV	April 20	Single group	None	- Oxygenation index (day 14)	10	18-75y	Severe pneumonia ARDS
NCT04264533	1. Vitamin C IV 2. Placebo	September 20	Randomized Parallel	Yes (PCOa)	- Ventilation-free days (day 28)	140	≥18y	Severe pneumonia ARDS
NCT04323527	1. Chloroquine (low) O 2. Chloroquine (high) O	August 20	Randomized Parallel	Yes (PCIOa)	- Absolute mortality (day 28)	440	18-100y	SARS with or without infection^*^
NCT04276688	1. Lopinavir/Ritonavir O + Ribavirin O + Interferon ß-1b SC 2. Lopinavir/Ritonavir O	January 22	Randomized Parallel	None	- Time to negative nasopharyngeal swab (month 1)	70	≥18y	Infection^*^
EudraCT-2020-001200-42	1. Camostat mesylate O 2. Placebo	na	Randomized Parallel	Yes (DB)	- Time to clinical improvement (from day 0 to discharge/death)	180	≥18y	Infection^*^
EudraCT- 2020-001023-14	1. Interferon β-1a IN 2. Placebo	na	Randomized	Yes (DB)	- Clinical improvement (day 14)	400	≥18y	Infection^*^
EudraCT-2020-001224-33	1. Hydroxychloroquine O 2. Placebo	na	Randomized Parallel	Yes (DB)	- Viral clearance (RT-PCR)	220	≥18y	Severe pneumonia
EudraCT-2020-001243-15	1. Itraconazole O 2. Best clinical practice	na	Randomized	None	- Clinical improvement (day 15)	200	≥18y	Pneumonia Severe pneumonia ARDS
Phase 2-3								
NCT04315298	1. Sarilumab (low) IV 2. Sarilumab (high) IV 3. Placebo	March 21	Randomized Parallel	Yes (PCIOa)	- Time to resolution of fever (day 29) - Clinical improvement (day 15)	400	≥18y	Severe pneumoniaARDS Sepsis
NCT04278963	1. Yinhu Qingwen Decoction (low) O 2. Yinhu Qingwen Decoction (high) O	January 21	Randomized Parallel	Yes (PO)	- Mean clinical recovery time (day 28)	300	≥18y	Pneumonia
NCT04275414	1. Bevacizumab IV	April 20	Single group	None	- PaO2 to FiO2 ratio (day 1,3,7)	20	18-80y	ARDS
NCT04322344	1. Escin O 2. Escin IV 3. Standard therapy	June 20	Non-randomized Parallel	Yes (PC)	- Mortality rate (day 30) - Clinical status (day 30)	120	18-75y	Infection^*^
NCT04323592	1. Methylprednisolone IV	May 20	Single group	None	- Death or ICU admission or Invasive ventilation (composite)(day 28) - Death (day 28) - Admission to ICU (day 28) - Endotracheal intubation (day 28)	104	18-80y	ARDS
NCT04244591	1. Methylprednisolone IV 2. Standard of care	April 20	Randomized Parallel	None	- Lower Murray lung injury score (day 7,14)	80	≥18y	ARDS
NCT04319900	1. Favipiravir O + Chloroquine O 2. Favipiravir O 3. Placebo	April 20	Randomized Parallel	Yes (PC)	- Time of improvement or recovery of respiratory symptoms (day 10) - Number of days virus nucleic acid shedding (day 10) - Frequency of improvement or recovery of respiratory symptoms (day 10)	150	18-75y	Pneumonia Severe pneumonia
EudraCT-2020-001246-18	1. Sarilumab IV 2. Tocilizumab IV 3. Anakinra IV4. Standard of care	na	Randomized Parallel	None	- Survival without needs of ventilator utilization (day 14) - Cumulative incidence of successful tracheal extubation (day 14) - Clinical improvement (day 4)	1,000	≥18y	Pneumonia Severe pneumonia ARDS
EudraCT-2020-001113-21	1. Lopinavir/Ritonavir O 2. Interferon β-1a IN 3. Dexamethasone IV4. Hydroxychloroquine O	na	Randomized Parallel	None	- In-hospital mortality (day 28)	2,000	≥18y	Severe pneumonia ARDS
EudraCT-2020-001162-12	1. Sarilumab IV 2. Placebo	na	Randomized Parallel	Yes (DB)	- Time to resolution of fever (day 29) - Clinical improvement (day 15)	460	≥18y	Severe pneumoniaARDS

*IM, intramuscular; IN, inhaled; IV, intravenous; O, oral; SC, subcutaneous; DB, double blind; P, participant; I, investigator; C, care provider; Oa, outcomes assessor*.

* *Not based on the WHO classification of COVID-19 associated conditions*.

**Figure 2:**
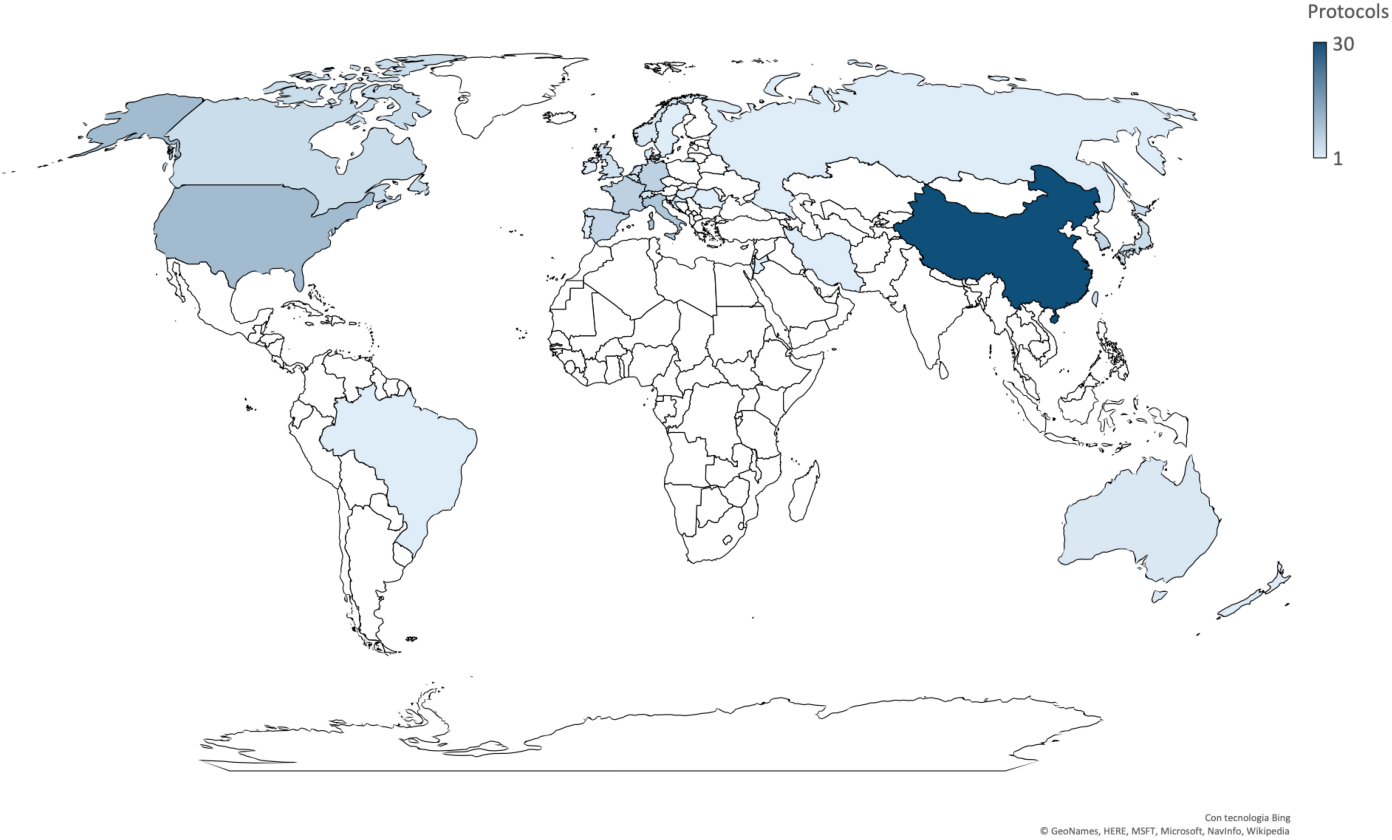
Geographic location of the selected protocols.

Most trials were conducted in China (*n* = 30), followed by the US (*n* = 10), Italy (*n* = 8), Germany (*n* = 6), France (*n* = 6), Spain (*n* = 5), and Korea (*n* = 5) (Figure 2). Seven trials involved international networks of clinical sites, whereas 55 were run in single countries; 35% of studies (*n* = 22) are multicentric. The majority of studies were funded by non-commercial research institutions (e.g., universities, hospitals, foundations, institutes) while only 11 were sponsored by the biopharma industry. Protocols had a varying duration and are expected to be completed (in terms of primary completion) between April 2020 and July 2023.

Forty-five trials had a randomized design, mostly relying on a parallel assignment of participants. Masking procedures were instead adopted by less than half of the trials (*n* = 26), with nine studies reporting quadruple masking involving both the participants, investigators, care providers, and outcomes assessors. Placebo or standard care were used as comparators in 27 studies, and 14 use one or more active comparators, whereas nine compared different regimens (i.e., dosages and/or duration) of the same treatment.

### Tested Interventions

**Figure 3:**
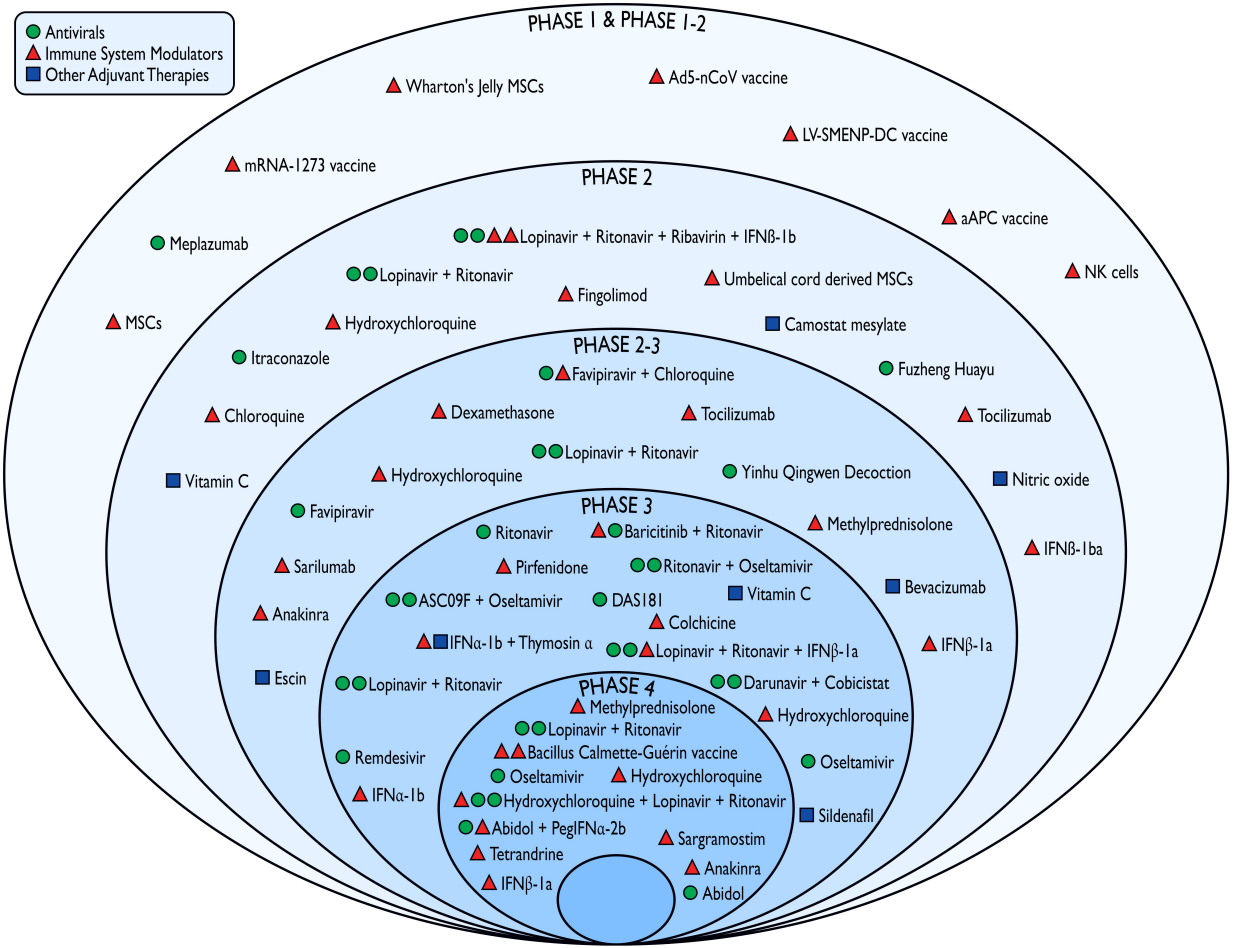
Pharmacological interventions against COVID-19 tested in the emergency phase of the outbreak according to the study phase and mechanism of action. IFN, interferon; MSCs, mesenchymal stem cells; NK, natural killers.

Most protocols (*n* = 32) investigated the efficacy and/or safety profiles of compounds that are expected to act as immune system modulators in COVID-19 associated conditions (Figure 3). These compounds included vaccines (*n* = 5), cell-based therapies (*n* = 6; e.g., mesenchymal stem cells, natural killer cells), antimalarial drugs (*n* = 9; e.g., chloroquine and hydroxychloroquine), corticosteroids (*n* = 4), interleukin inhibitors, and interferons. Twenty-two studies have been testing antiviral agents such as antiretroviral protease inhibitors (e.g., darunavir, lopinavir, ritonavir), neuraminidase inhibitors (e.g., oseltamivir), nucleotide analogs (e.g., remdesivir), and broad-spectrum antivirals. The remaining trials were designed to investigate other potential adjuvant therapies such as nitric oxide, antioxidants, phosphodiesterase inhibitors. Finally, seven studies have been evaluating the combinations of substances with both immunomodulant and antiviral properties.

Most of the selected primary outcome measures referred to clinical endpoints (e.g., mortality rates, clinical improvement/remission, hospital discharge, intensive care unit admission, ventilation-free days). A sizeable proportion of studies (*n* = 24) incorporated laboratory (e.g., viral clearance/load) or radiological (e.g., change of pneumonia severity on X-ray or CT) changes as primary endpoints. A residual number of trials (*n* = 7) were instead primarily aimed at exploring the safety and tolerability profiles of the tested interventions.

### Targeted Conditions

A total of 41,110 participants will tentatively be recruited in the selected protocols, with sample sizes widely ranging between five and 6,800 subjects.

The entire clinical spectrum of COVID-19, ranging from infection with mild symptoms to sepsis complicated by shock, was targeted by the studies in the emergency phase of the COVID-19 outbreak. It also planned to recruit healthy subjects or individuals exposed to higher risk (e.g., healthcare providers or household contact).

Early phase studies (Table 1) preliminarily tested the tolerated dose, the safety, and efficacy of candidate agents in small representative groups. The target population was composed by healthy subjects (*n* = 4), individuals with laboratory confirmed infection without a clear WHO definition of the clinical syndrome (*n* = 6), patients with pneumonia ranging from mild to severe (*n* = 19), and more severe/critical clinical syndromes including acute respiratory distress syndrome (ARDS) and sepsis (*n* = 14). In the late phases studies (Tables 2, 3) that are testing on a large scale those agents with documented safety and evidence of preliminary efficacy in the earlier phases, the target participants were largely represented by healthy or at-risk subjects with infection (*n* = 7), patients with mild/severe pneumonia (*n* = 20), and patients with more severe/critical clinical syndrome (*n* = 10).

**Table 2:** Characteristics of the selected phase 3 protocols targeting COVID-19-related conditions.

**Identification trial number**	**Treatment(s) Comparator(s)(if any)**	**Primary completion**	**Allocation Assignment**	**Masking**	**Primary outcome(s)**	**Subjects**	**Age**	**Condition**
NCT04292899 EudraCT-2020-000841-15	1. Remdesivir IV (5 days) 2. Remdesivir IV (10 days)	May 20	Randomized Parallel	None	- Proportion with normalization of fever and oxygen saturation (day 14)	2,400	≥18y≥12y	Pneumonia
NCT04292730 EudraCT-2020-000842-32	1. Remdesivir IV (5 d) 2. Remdesivir IV (10 d) 3. Standard of care	May 20	Randomized Parallel	None	- Proportion of participants discharged by (day 14)	600	≥18y	Pneumonia
NCT04304313	1. Sildenafil O	Mar 20	Single group	None	- Rate of disease remission (day 14) - Rate of entering the critical stage (day 14) - Time of entering the critical stage (day 14)	10	≥18y	Pneumonia Severe pneumonia
NCT04304053	1. Darunavir/Cobicistat O 2. Hydroxychloroquine O 3. Isolation	June 20	Cluster-RCT Randomized Parallel	None	- Incidence of secondary COVID-19 cases among contacts (day 14) (chemoprophylaxis)	3,040	≥18y	Healthy subjects Infection^*^
NCT04252664	1. Remdesivir O 2. Placebo	April 20	Randomized Parallel	Yes (PCIOa)	- Time to clinical recovery (day 28)	308	≥18y	Pneumonia
NCT04320238	1. Recombinant human Interferon α-1b IN 2. Recombinant human Interferon α-1b IN + Thymosin α1 SC	May 20	Non-randomized Parallel	None	- New-onset COVID-2019 (week 6)	2,944	18–65y	Healthy health care providers
NCT04261270	1. ASC09F + Oseltamivir O 2. Ritonavir + Oseltamivir O 3. Oseltamivir O	May 20	Randomized Parallel	Yes (P)	- Rate of comprehensive adverse outcome (day 14)	60	18–55y	Pneumonia
NCT04322682	1. Colchicine O 2. Placebo	September 20	Randomized Parallel	Yes (P)	- Composite of death or the need for hospitalization due to COVID-19 infection (day 30)	6,000	≥40y	Infection^*^
NCT04315948 EudraCT-2020-000936-23	1. Remdesivir IV 2. Lopinavir/Ritonavir O 3. Lopinavir/Ritonavir O + Interferon β-1a SC 4. Hydroxychloroquine O 5. Standard of care	March 23	Randomized Parallel	None	-Clinical improvement (day 15)	3,100	≥18y	Pneumonia Severe pneumonia ARDS
NCT04280705 EudraCT-2020-001052-18	1. Remdesivir IV 2. Placebo	April 23	Randomized Parallel	Yes (PI)	-Clinical improvement (day 15)	440	18–99y	Pneumonia Severe pneumonia ARDS
NCT04257656	1. Remdesivir IV 2. Placebo	April 20	Randomized Parallel	Yes (PCIO)	- Time until clinical improvement (day 28)	453	≥18y	Severe pneumonia ARDS
NCT04252274	1. Darunavir/Cobicistat O 2. Conventional treatment	August 20	Randomized Parallel	None	- Virological clearance (day 7)	30	All	Pneumonia Severe pneumonia
NCT04320277	1. Baricitinib + Ritonavir O 2. Ritonavir O and/or Hydroxychloroquine O	April 20	Non-randomized Crossover	None	- Percentage of ICU admission in patients vs. controls (week 2)	60	18-85y	Pneumonia
NCT04308668	1. Hydroxychloroquine O 2. Placebo	April 20	Randomized Parallel	Yes (PCIOa)	- Incidence of COVID-19 in asymptomatic subjects (day 14) - Change in COVID-19 Severity (day 14) among symptomatic:	3,000	≥18y	Healthy subjects Infection^*^
NCT04282902	1. Pirfenidone O 2. Standard of care	April 20	Randomized Parallel	None	- Laboratory, imaging and clinical improvement (week 4)	294	≥18y	Severe pneumonia
NCT03680274	1. Vitamin C IV 2. Placebo	December 21	Randomized Parallel	Yes (PCIOa)	- Deceased participants or with persistent organ dysfunction (day 28)	800	≥18y	Sepsis
NCT03808922	1. DAS181 IN 2. Placebo	April 21	Randomized Parallel	Yes (PCIO)	- Clinical status improvement (day 14) (sub-study)	250 (main study)	All	Severe pneumonia
EudraCT-2020-000982-18	1. Hydroxychloroquine O 2. Standard of care	na	Randomized	None	- In-hospital mortality	443	≥18y	Severe pneumonia ARDS Sepsis/septic shock
EudraCT-2020-000890-25	1. Hydroxychloroquine O	na	Single group	None	- Results of SARS-CoV2 virus detection (day 1,4,7,14)	25	≥12y	Infection^*^

*IN, inhaled; IV, intravenous; O, oral; SC, subcutaneous; P, participant; I, investigator; C, care provider; Oa, outcomes assessor*.

* *Not based on the WHO classification of COVID-19 associated conditions*.

**Table 3:** Characteristics of the selected phase 4 protocols targeting COVID-19-related conditions.

**Identification trial number**	**Treatment(s) Comparator(s)(if any)**	**Primary completion**	**Allocation Assignment**	**Masking**	**Primary outcome(s)**	**Subjects**	**Age Years**	**Condition**
NCT04308317	1. Tetrandrine O 2. Standard of care	March 21	Randomized Parallel	None	- Death event (week 12)	60	18–75y	PneumoniaSevere pneumonia
NCT04326920 EudraCT-2020-001254-22	1. Sargramostim IN or IV 2. Placebo	October 20	Randomized Parallel	None	- Improvement in oxygenation (day 5)	80	18–80y	Severe pneumonia ARDS
NCT04255017	1. Abidol hydrochloride O 2. Oseltamivir O 3. Lopinavir/Ritonavir O 4. Symptomatic treatment	June 20	Randomized Parallel	Yes (P)	- Rate of clinical remission (week 2) - Time of lung imaging recovery (week 2)	400	≥18y	PneumoniaSevere pneumoniaARDS
NCT04254874	1. Abidol hydrochloride O 2. Abidol hydrochloride O + Interferon (PegIFNα-2b) IV	June 20	Randomized Parallel	Yes (P)	- Rate of clinical remission (week 2) - Time of lung imaging recovery (week 2)	100	≥18y	Pneumonia Severe pneumonia ARDS
NCT04263402	1. Methylprednisolone (<40mg) IV 2. Methylprednisolone(40-80mg) IV	June 20	Randomized Parallel	Yes (P)	- Rate of disease remission (day 7) - Rate and time of entering the critical stage (respiratory failure or multiorgan failure)(day 7)	100	≥18y	Severe pneumonia ARDS
NCT02735707	1. No antiviral 2. Lopinavir/Ritonavir O 3. Hydroxychloroquine O 4. Hydroxychloroquine O + Lopinavir/Ritonavir O 5. No immune modulators 6. Interferon β-1a IV 7. Anakinra IV	December 21	Randomized Factorial	None	- All cause death (day 90) - Days alive and outside of ICU (day 21)	6,800 (main study)	≥18y	Sever pneumoniaARDSSepsis/septic shock
NCT04252885	1. Lopinavir/Ritonavir O 2. Arbidol O 3. Standard of care	May 20	Randomized Parallel	None	-The rate of virus inhibition (in nose/throat swab) (day 0,2,4,7,10,14,21)	125	18–80y	Infection^*^
2020-001010-38	1. Hydroxychloroquine O 2. Standard of care		Randomized	None	-Rate of decline in SARS-CoV-2 viral load in nasopharyngeal samples (96 h)	200	≥18y	Pneumonia
EudraCT-2020-000919-69	1. Bacillus Calmette-Guérin vaccination ID		Randomized	Yes (DB)	- Number of days of unplanned absenteeism for any reason (hospital personnel) (days 0–180)	1,000	≥18y	Healthcare providers

*ID, intradermal; IN, inhaled; IV, intravenous; O, oral; DB, double blind; P, participant*.

* *Not based on the WHO classification of COVID-19 associated conditions*.

## Discussion

Although the first COVID-19 cases were reported just 4 months ago (11), there has been an unprecedented response from the international community. The findings of several interventional studies have already been disseminated (5, 12). Encouragingly, a relevant number of clinical trials have explored safe and effective therapeutics to face the pandemic, enrolling individuals with COVID-19 worldwide, and some of these trials will publish the results as early as in the next few weeks/months. This emerging evidence will largely be concerned with hard outcomes such as mortality (adopted as the primary endpoint in ten studies), access to intensive care units, clinical remission, and will therefore have profound clinical implications.

This review provides an overview of studies in the emergency phase of the outbreak, utilizing the two most common open access protocol registries in the US and Europe, with the aim of informing clinicians and researchers on what they can expect in the upcoming months. Of note, is the fact that we restricted our focus to only two clinical trial registries and we are aware that this might potentially underestimate the current situation. When a broader search is conducted by including most of the existing national and international databases, the number of ongoing studies is much higher and needs to be constantly updated (13). Accordingly, coalitions/networks have recently been launched to provide frequently updated resources (e.g., living systematic reviews) summarizing the characteristics of research protocols targeting COVID-19 (7, 13). These initiatives are particularly welcomed, as they potentially allow for the coordination of a multinational research effort and better allocation of the available research resources.

As expected, interventional studies were largely performed and promoted in those countries where the outbreak has already significantly affected the community and the healthcare system. The inclusion criteria of the studies was designed to target the entire spectrum of clinical syndromes associated with COVID-19 at the time the study was conducted, namely asymptomatic status, mild illness, pneumonia, ARDS, and septic complications. The opportunity to include the clinical struggles for different categories of patients was also implemented. Several trials were instead focused on the vaccination and chemoprophylaxis of healthy individuals. Two studies were specifically dedicated to health care providers, consistently with their established vulnerability in the COVID-19 pandemic (14). These studies are very much needed, as in some countries the number of healthcare providers with infection is rapidly increasing due to a shortage of personal protective equipment, in parallel with the high demand for care that usually occurs during a pandemic (15). Currently, slowing the spread of the SARS-CoV-2 relies on measures of social distancing and recommended changes to lifestyle and behavior that have unmeasurable consequences on the life of individuals and communities, not to mention the economic crisis that countries face. In light of this, it is pivotal to cooperate and optimize the effort for a common solution starting from the systematic recruitment of patients to complete the ongoing trials.

Based on the registered information, some protocols will probably provide *proof-of-concept* evidence supporting the design of large-scale clinical trials. Conversely, some of the ongoing phase 3 randomized controlled trials and phase 4 post marketing studies seem already adequately informed to be able to draw either positive or negative conclusions on the efficacy and safety/tolerability of pharmacological compounds with different mechanisms of action. Of note, is the fact that some trials are adopting adaptive designs, allowing them to rapidly accept or reject multiple experimental therapies, which is especially promising in the current outbreak scenario (4).

The major limitation of our study is related to the extremely dynamic evolution of knowledge on the topic. As mentioned, an incredible number of trials have been proposed on COVID during the past weeks and it is likely that this number will rapidly and exponentially increase in the next months, especially given the more consistent dissemination of the coronavirus in different regions of the world. In this regard, since April 1, 2020, 585 new protocols have been registered on the clinicaltrials.gov database (search updated to August 18, 2020) with an expected overall number of around 375,000 participants. As compared with the emergency phase, a greater proportion of phase 1 and 2 studies are currently active (70.2 vs. 54.8%). An increase in the percentage of industry-funded trials (34.2 vs. 17.7%) and of studies adopting a randomized design (85.3 vs. 72.6%) has been observed. As expected by the changes that have occurred in the epidemiology of COVID-19, the US and Europe persist as the main recruiting sites while centers in South America, India, and Africa have recently started to contribute. It is noteworthy that, due to a better understanding of the pathophysiological mechanisms of the disease (10), there are a relevant number of novel compounds, mostly acting as immunomodulators, that are being tested (e.g., ruxolitinib, colchicine, heparins, mavirilumab, ivermectin). These were not present nor in the pipeline at the end of March 2020. Moreover, 47 protocols are currently investigating the efficacy and safety profiles of vaccines whereas 67 focused on convalescent plasma therapies. As of August 18, 2020, the (negative) findings of four of the studies that started in the emergency phase have already been published (i.e., three testing remdesivir [NCT04292899, NCT04292730, NCT04257656] and one testing hydroxychloroquine [NCT04308668]) (16–19).

In conclusion, the present analysis provides an account for researchers and clinicians for them to understand present research and envision the future of therapeutics testing for the management of the COVID-19 pandemic.

## Data Availability Statement

All datasets presented in this study are included in the article.

## Author Contributions

MCa and VR: study design, data analysis, and writing of the manuscript. GRe: study design, data collection, and drafting of the manuscript. FT, GRic, LT, GRis, and AA: data collection. GB, MCe, and NV: data interpretation and drafting of the manuscript. All authors contributed to the article and approved the submitted version.

## Conflict of Interest

The authors declare that the research was conducted in the absence of any commercial or financial relationships that could be construed as a potential conflict of interest.
